# Three dimensional Phase Sensitive Inversion Recovery (PSIR) turbo FLASH for evaluation of left ventricular myocardial lesions in infiltrative and non-ischemic cardiac diseases

**DOI:** 10.1186/1532-429X-11-S1-P200

**Published:** 2009-01-28

**Authors:** Aya Kino, Sven Zuehlsdorff, Aoife Keeling, Cormac Farrelly, John Sheehan, Randall Ramsay, Renate Jerecic, James C Carr

**Affiliations:** 1 Northwestern University, Chicago, IL USA; 2 Siemens Medical Solutions USA, Inc., Chicago, IL USA

**Keywords:** Gadopentetate Dimeglumine, Image Quality Score, Myocardial Lesion, Phase Sensitive Inversion Recovery, Isotropic Coverage

## Background

Delayed-enhanced MRI (DE-MRI) is typically used to assess myocardial viability by depicting regions of myocardial scarring caused by myocardial infarction. Infiltrative myocardial disease and non-ischemic cardiomyopathy demonstrate atypical patterns of enhancement on DE-MRI, producing diffuse hyperenhanced lesions throughout the myocardium. Such hyperenhanced lesions patterns may be missed by conventional 2D imaging due to non-contiguous slices and limited slice coverage. 3D DE-MRI may be a more effective technique for assessing hyperenhanced lesions due to the isotropic coverage of left ventricle (LV).

## Purpose

The purpose of this study was to compare a navigator gated free breathing 3D Phase Sensitive Inversion Recovery (PSIR) turboFLASH to an established 2D PSIR turboFLASH method for detecting myocardial hyperenhanced lesions caused by infiltrative myocardial disease and non-ischemic cardiomyopathy.

## Materials and methods

Under an IRB approved protocol, 18 patients with suspected infiltrative myocardial heart disease and cardiomyopathy [hypertrophic cardiomyopathy (HCM) n = 9, sarcoidosis, n = 4, and myocarditis, n = 5] were examined on a 1.5 T MR scanner (MAGNETOM Avanto, Siemens AG, Erlangen, Germany) after the administration of gadopentetate dimeglumine [0.2 mmol/kg Gadolinium-DTPA (Magnevist, Schering AG, Berlin, Germany)] using a segmented 2D PSIR turboFLASH sequence and a navigator-gated 3D PSIR turboFLASH sequence. Quantitative evaluation was carried out by measuring the volume of hyperenhaced lesions for both techniques using the VPT quantification software (Siemens AG) that considered areas with 6 standard deviations above the normal myocardial signal intensity as abnormal. Images were assessed qualitatively by 2 reviewers using the AHA 17-segment model. The presence and transmural extent of lesions were evaluated and the number of lesion per segment was counted. Image quality was scored using a four point Likert scale (0-poor, non-diagnostic; 1-fair, diagnostic maybe be impaired; 2-good with some artifacts and 3-excellent without artifacts).

## Results

Average total scan time for the 18 patients was 9:56 minutes for 2D PSIR and 6:54 minutes for 3D PSIR, respectively. The mean navigator efficiency was 53.2%.

**Figure 1:**
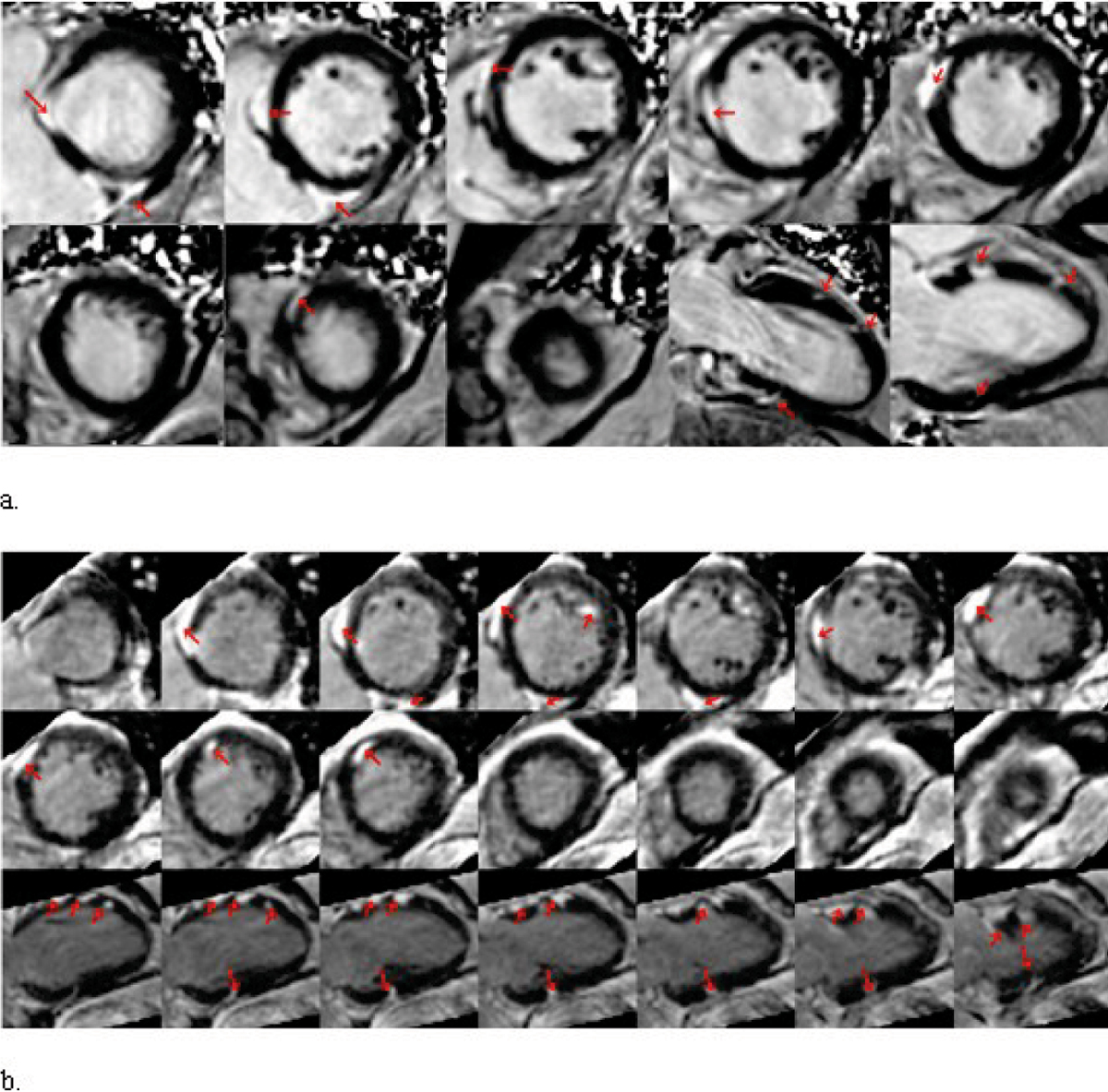
**(a) 2D PSIR images of the left ventricle of a 55-year-old male patient with biopsy proven sarcoidosis showing multiple scattered hyper enhanced lesions** ***(red arrows)*** **within the left ventricular myocardium in short axis, 2 and 3 chamber views**. **(b)** 3D PSIR reformatted images from the same patient demonstrate more numerous lesions in a subepicardial and mid myocardial location *(red arrows)* compared to 2D PSIR.

The image quality score did not differ significantly (p = 0.53) for both techniques. The total number of hyperenhanced regions detected using 3D PSIR was larger than at 2D PSIR (p < 0.05) (Figure 1). Qualitative analysis of area of lesions (p = 0.15) and location (p = 0.38) were similar for both techniques.

## Conclusion

Free breathing 3D PSIR viability imaging may be more effective than conventional 2D imaging for detecting hyperenhaced lesions associated with infiltrative heart disease and non-ischemic cardiomyopathy. The 3D approach provides an isotropic coverage of the LV and detects smaller lesions missed by 2D imaging.

## Abstract Summary Statement

Comparison between free breathing 3D Phase Sensitive Inversion Recovery (PSIR) turboFLASH and 2D PSIR turboFLASH for detecting myocardial lesions in infiltrative and non-ischemic cardiac diseases. 3D PSIR is a promising alternative for quantification and detection of small and diffuse lesions.

